# Blunt Force Trauma in the Canarian Houbara Bustard (*Chlamydotis undulata fuertaventurae*) Produced by Collision with Overhead Lines

**DOI:** 10.3390/vetsci11070287

**Published:** 2024-06-27

**Authors:** Cristian M. Suárez-Santana, Lucía Marrero-Ponce, Jose Navarro-Sarmiento, Candela Rivero Herrera, Raiden Grandía-Guzmán, Ana Colom-Rivero, Óscar Quesada-Canales, Eva Sierra, Antonio Fernández

**Affiliations:** Unit of Veterinary Histology and Pathology, University Institute of Animal Health and Food Safety (IUSA), Veterinary School, University of Las Palmas de Gran Canaria (ULPGC), 35413 Las Palmas de Gran Canaria, Canary Islands, Spain; cristian.suarez@ulpgc.es (C.M.S.-S.); eva.sierra@ulpgc.es (E.S.); antonio.fernandez@ulpgc.es (A.F.)

**Keywords:** houbara bustard, trauma, cause of death, collision, overhead line, forensic necropsy, bird population decline

## Abstract

**Simple Summary:**

Bird mortality resulting from collisions and electrocutions with overhead lines (such as power lines and phone lines) has contributed to the decline of various avian species globally. Specifically, overhead line collisions pose a significant threat to the conservation of the Canarian houbara bustard (Chlamydotis undulata fuertaventurae), an endangered subspecies endemic to the Canary Islands. This study focuses on postmortem findings from Canarian houbara bustards that collided with overhead lines. We describe the necropsy findings of Canarian houbara bustards found dead near overhead lines, and we investigated, using a forensic approach, whether the animal survived the initial trauma.

**Abstract:**

The mortality of birds resulting from collisions and electrocutions with overhead lines, such as power lines and phone lines, among others, has been implicated in the decline of various avian species globally. Specifically, overhead line collisions pose a significant threat to the conservation of the Canarian houbara bustard (*Chlamydotis undulata fuertaventurae*), an endangered subspecies endemic to the Canary Islands. This study centers on the postmortem findings of Canarian houbara bustards that have collided with overhead lines, providing insights into the post-collision outcomes for these birds. A complete standardized necropsy of nine Canarian houbara bustards revealed that trauma was the cause of death in all cases. The most notable gross lesions associated with trauma included bone fractures, soft tissue lacerations, hemorrhages, luxations, and hemocoelom. The inguinal area, chest, and wings were the body regions more frequently affected. A histopathology, immunohistochemistry, and entomology analysis confirmed that numerous birds survived the initial trauma. We concluded that when a houbara bustard collides with an overhead line, it frequently survives the initial trauma, with a survival time ranging from minutes to hours. The histopathology, immunohistochemistry, or entomologic analysis may be helpful to approximate the timing interval between trauma and death.

## 1. Introduction

The mortality of birds due to collision with overhead lines (i.e., power lines, phone lines, telegraph wires, etc.) and electrocution has been linked to the decline of diverse avian species around the world [1,2,3,4]. However, most studies report the estimation of mortalities due to power line collisions by documenting the presence of carcasses along the electric or communication lines, and the confirmation of trauma with standardized necropsies are not commonly performed [2,3,4,5,6]. If the initial suspicion of collision is not confirmed with necropsy, other causes of death, such as electrocution, can be overlooked [7].

Although overhead lines constitute a possible hazard for all birds during flight, certain species, such as the bustards, tend to be predisposed to collision [1,8,9].

The Canarian houbara bustard (*Chlamydotis undulata fuertaventurae*) is an endemic subspecies of houbara bustard located in Fuerteventura, Lanzarote, and La Graciosa in the Canarian Archipelago (Spain) [10], that has been closely monitored during the last years by scientists [1,10,11]. This subspecies is considered “in danger” on the Spanish Red List [11] and as “vulnerable” by the IUCN (2009) [12], with an estimated population of 525–750 individuals according to different authors [1,11,13,14]. Power line collisions have been recognized as a significant problem for the conservation of these animals [1,6], and recent investigations predict the extinction of the subspecies in 50 years due to anthropogenic mortality [1].

Legislation in different parts of the world may protect endangered species, and when an overhead line is proved to cause repetitive episodes of mortality, corrective measures must be taken by the owner of problematic overhead lines. Forensic veterinary reports are usually determinant in the judicial process, and to prove a case of collision, there must be clear evidence of blunt force trauma [15,16]. Moreover, it should be noted that collision with overhead lines is not the only source of trauma for birds, and that an injured bird is more likely to suffer subsequent traumas, such as interactions with other animals (predation) or being hit by a car. These other secondary traumatisms may mislead the forensic veterinarian who is trying to answer the questions of why and how this animal died.

In this study, we focus on the lesions observed in houbara bustards that collided with overhead lines and try to determine if the houbara survived the initial trauma.

## 2. Materials and Methods

This work is part of a more extensive survey of the causes of mortality in the Canarian wildlife created by the Canarian Government, and is known as Red Vigía Canarias (Orden Nº134/2020 de 26 de mayo de 2020).

Inclusion criteria for the study were (1) Animal identified as Canarian houbara bustard; (2) Blunt force trauma as the cause of death; and (3) History suggests collision with an overhead line. Very decomposed animals and animals without a known history were excluded.

A complete necropsy was performed in all cases following standardized procedures [17]. Whole body radiographs were obtained from each individual before necropsy. Feathers were inspected and removed entirely to allow for the observation of the skin and to evaluate for signs of electrocution. Nutritional condition was evaluated by assessing the pectoral muscle mass (keel score) and the presence of fat, and classified in a numeric scale as 1 to 5 (1 cachexia, 2 thin, 3 normal, 4 overweight, 5 obesity) (modification of the body score presented by Burton et al., 2014 [18]). Animals’ age was estimated and included in one of the following categories according to body morphology and skeletal and gonadal development: chick, juvenile or adult.

Samples for histopathology included adrenal glands, encephalon, esophagus, eyes, heart, intestine, kidney, liver, lung, pancreas, spleen, stomach, testicles, thyroid, tongue, and trachea, which were collected and placed in 10% buffered formalin for 24 h and routinely processed. Additionally, when traumatic lesions were observed in any other body location, additional tissues, including skin, muscle, and bone, were collected and processed.

Histochemistry was performed on selected tissue sections, including Periodic Acid Schiff (PAS), phosphotungstic acid hematoxylin (PTAH) and Prussian blue. Chromic acid technique was performed to fix the lipids to the tissues before paraffin embedding, and the lung sections were later stained with Oil Red O [19]. PTAH was used to highlight changes indicative of acute muscle degeneration, whereas Prussian blue was used to detect ferric ions indicative of hemosiderin byproducts.

Immunohistochemistry against myoglobin and fibrinogen was performed in Cases 6, 7 and 9 using a previously published methodology [20,21,22]. Briefly, 3 µm thick sections were deparaffined, rehydrated, and labelled with polyclonal anti-myoglobin and anti-fibrinogen antibodies (Abcam, Cambridge, UK); the secondary antibody consisted of polyclonal swine anti-rabbit immunoglobulin (Dako, Glostrup, Denmark), and the antibodies were visualized using the VECTASTAIN Elite ABC-Peroxidase kit (PK-6100, Vector Laboratories, Peterborough, UK). The negative control followed the same methodology, but primary antibodies were omitted. The positive control for myoglobin included the skeletal muscle of each case (internal control) and skeletal muscle from a dolphin with rhabdomyolysis (external control) [21]. The positive control of fibrinogen was the liver of a live-stranded dolphin with extensive hepatocyte vacuolar degeneration [20,22]. For more details of the immunohistochemical method, see the Supplementary Materials.

Diptera eggs and larvae were collected from Case 5 during the necropsy. The eggs were processed for histology and part of them were cultivated inside a sterile container and fed with meat at 25 °C for 5 days [23].

## 3. Results

Between 2020 and 2023, 98 Canarian houbara bustards were submitted for necropsy. In 15 animals, the cause of death was determined as blunt force trauma. The history of nine houbara bustards indicates a collision with an overhead line as the etiology of trauma (Cases 1 to 9, see Table 1). Of these, eight were submitted from Lanzarote, and one was submitted from Fuerteventura. All the animals were adults (i.e., sexually mature). Five were male, four were female, and in one case, the sex could not be determined due to predation (Case 8). The state of decomposition was established during necropsy, resulting in seven animals being fresh, and two carcasses were incipiently decomposed. Seven of the nine animals ported a GPS transmitter attached to their back with elastic bands, included in another study performed by the Natural Sciences History Museum of Madrid (CSIC) [1].

Two cases were found under a high-voltage power line, whereas the remaining animals were found near medium-voltage electric lines or phone lines (Case 2). The distance from the lines ranged from 0 to 400 m. For more details of the GPS track information, see Alonso et al. (2024) [1].

### 3.1. Gross Lesions

Lesions associated with blunt force trauma were observed in all the animals and summarized in Table 1. In the following paragraphs we describe the most significant traumatic lesions and body regions affected.

**Figure 1:**
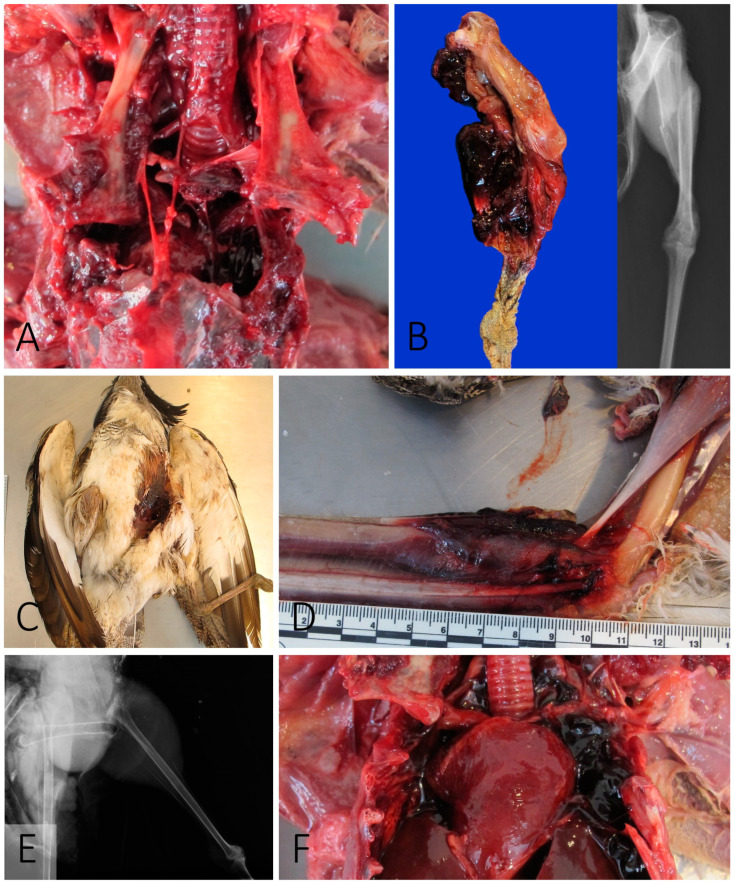
Macroscopic findings. (**A**) Case 9. Avulsive fracture of the sternum. (**B**) Case 1. Fracture of the tibiotarsus. (**C**) Case 5. Inguinal skin abrasion and laceration. (**D**) Case 9. Focally extensive hematoma in the left forearm. (**E**) Case 5. Knee luxation (radiography). (**F**) Case 9. Hemocoelom.

Bone fractures were observed in most cases (6/9). Fractures in the furcula were noted in four animals. Two affected the left side (Cases 2 and 4), and the other were bilateral (Cases 6 and 9). Fractures of the sternum were observed together with the furcular fracture in Cases 6 (Figure 1A) and 9. The left humerus was fractured in Case 2, whereas in Case 1, a multiple fracture of the diaphysis of the left tibiotarsus was noted (Figure 1B). In all cases, the fractures were associated with different degree of hemorrhage, ranging from very mild in the furcular fractures to extensive hematomas in the tibiotarsal fracture. As an incidental finding, Case 3 presented a healed keel fracture. More than one bone was fractured in 44.4% (*n* = 4) of the animals.

Skin and muscle lacerations were observed in five of the nine animals. In Cases 2 and 3, the left pectoral and inguinal regions had skin lacetarion with multiple rupture of muscle fibers. In Case 5, the skin laceration was in the left inguinal region (Figure 1C), whereas in Case 4 and 9, the pectoral and axillar regions were involved.

Hemorrhages indicative of recent trauma were detected in all cases. The location of the hemorrhages frequently coincides with the areas of bone fractures and skin lacerations. Extensive muscle hemorrhages were associated with bone fractures (Case 1), luxations (Case 5), and GPS elastic bands (Cases 7 and 9).

Luxations were observed in two cases. Luxation of the left knee, with severe focally extensive acute hematoma and left inguinal laceration, was noted in Case 5 (Figure 1E). Case 7 presented a bilateral coracoid luxation with an avulsive fracture of the body of the sternum.

Fatal internal bleeding was noted in four cases. Three of the animals developed hemocoelom (Cases 4, 6 and 9) (Figure 1F), whereas Case 7 had a severe hematoma between the musculus pectoralis and musculus supracoracoideus of the left side.

With regard to the affected body region, in most cases, the trauma was located at the inguinal region (7/9), followed by the chest (4/9), the wings (3/9), the shoulder (3/9), and the neck (3/9). Head trauma was not reported in any case.

Other non-traumatic gross findings included moderate to severe intestinal cestode parasitism in six animals (Cases 3, 4, 5, 7, 8 and 9) and moderate generalized muscle and fat atrophy (indicative of poor nutritional condition) in Cases 1 and 4.

### 3.2. Histopathology

**Figure 2:**
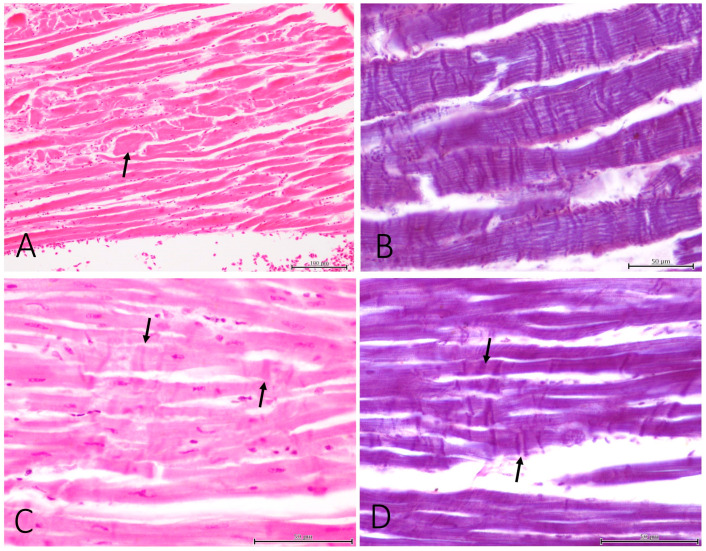
Histopathological findings. (**A**) Case 5. Quadriceps femoris muscle. Segmental necrosis of myocytes (arrow). (**B**) Case 9. Pectoral muscle. Contraction band necrosis (PTAH). (**C**,**D**) Case 5. Acute myocardial degeneration (arrows) ((**C**), HE; (**D**), PTAH).

Histopathology was performed on eight of the nine animals. The most commonly injured tissue was skeletal muscle, and the most representative traumatic lesions were acute skeletal muscle degeneration and segmental necrosis (Figure 2A). These lesions were noted in six cases in the pectoral muscle (Cases 1, 2, 3, 4, 7 and 9), and in the quadriceps femoris in Case 5 (Figure 2B). Muscle hemorrhages were also reported in the pectoral muscle of three cases (Case 2, 7 and 9) and the quadriceps femoris of Case 5. Cases 7 and 5 presented macrophages associated with necrotic muscle fibers, which indicates the lesions were at least a few hours old.

Acute myocardial degeneration and necrosis were observed in four cases (Cases 1, 2, 5 and 9). Degeneration consisted of nuclear pyknosis with hyperacidophilia, hypercontraction bands (Figure 2C,D) and the vacuolization of the sarcoplasm.

Severe pulmonary hemorrhages and edema were reported in five animals. Other lung changes included marked congestion (Cases 5, 7 and 9) and atelectasis (Case 9). Additionally, fat embolism was investigated in three animals (Cases 1, 2 and 3) using Oil Red O, but embolisms were not detected in the sections analyzed.

### 3.3. Immunohistochemistry

**Figure 3:**
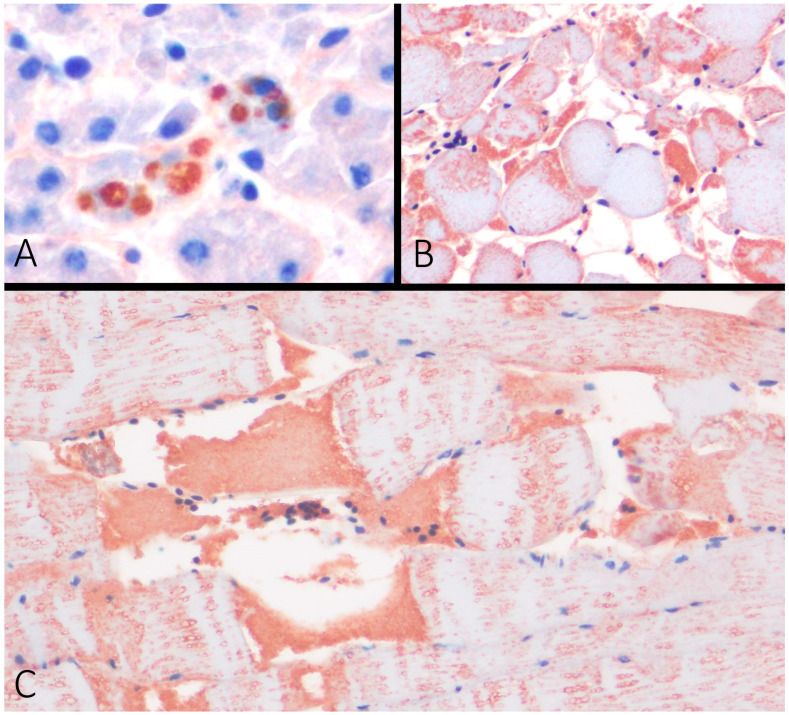
Immunohistochemistry for fibrinogen (**A**,**B**) and myoglobin (**C**). (**A**) Case 9. Intracytoplasmic vacuoles in hepatocytes are immunoreactive for fibrinogen. (**B**) Case 6. Skeletal muscle (pectoral) with degenerated myocytes is immunoreactive for fibrinogen. (**C**) Case 9. Skeletal muscle (pectoral) with segmental depletion of myoglobin and extracellular deposition of myoglobin.

Fibrinogen immunohistochemistry revealed immunopositivity in the intracytoplasmic vacuoles of the hepatocytes of Case 9 (Figure 3A), as well as strong intrasarcoplasmic and sarcolemma immunostaining in the degenerated myocytes of the skeletal muscle of Cases 6 and 7 (Figure 3B). Myoglobin immunohistochemistry revealed intrasarcoplasmic fibrillar staining of the cardiac and skeletal myocytes (internal positive control), but degenerated skeletal myocytes showed the segmental depletion of myoglobin and the extracellular deposition of myoglobin globules (Figure 3C). Myoglobin was not detected in the kidney in all cases (internal negative control). External positive and negative controls stained as expected.

### 3.4. Entomology

**Figure 4:**
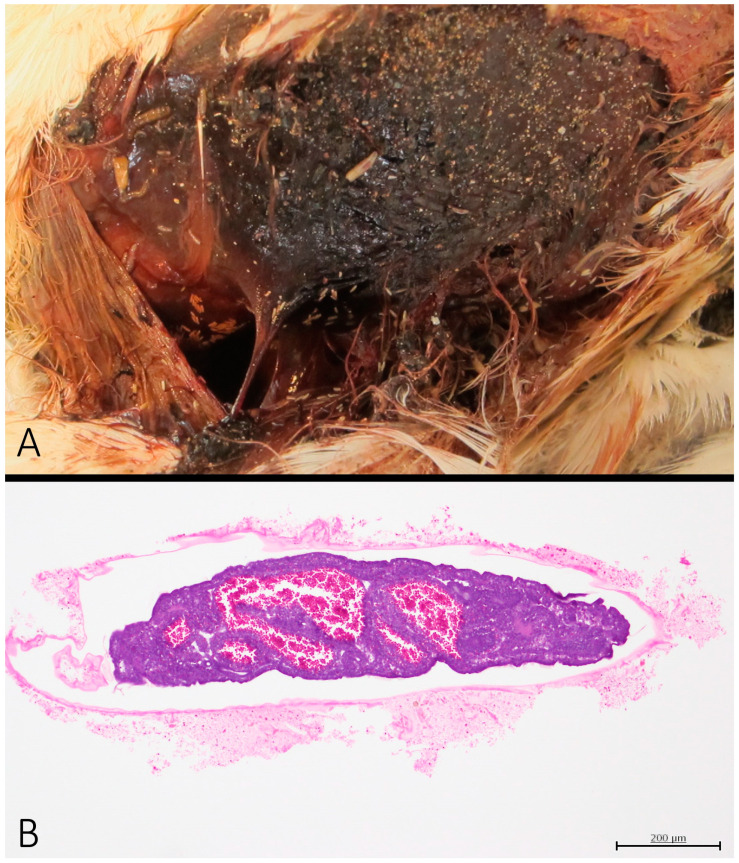
Entomology. (**A**) Case 2. Presence of larvae of diptera at various stages of development over a lacerated skin area. (**B**) Case 5. Diptera egg processed for histopathology shows an embryo with early development of digestive structures (HE).

Cases 2 and 5 had subcutaneous myiasis, with numerous eggs and larvae of diptera ranging in length from 3 to 13 mm (Figure 4A). The histology of the diptera eggs of Case 5 showed that most embryos had development of the proctodeum and stomodeum (Figure 4B), whereas few of them presented full development, which is consistent with embryos between 12 and 24 h of development [23]. Three mm larvae hatched after 24 h of egg culturing. After 48 h, only 13 mm larvae remained in the container, and larger larvae were not observed after an additional 72 h. These results indicate that 3, 8 and 13 mm larvae correspond to instar I, II and III, and that III instar develops after 72 h.

The entomology data, in association with the GPS information and the fresh status of the carcass, confirms that the animal collided with the overhead line and survived at least 24 h [24]. By that time, numerous flies had deposited eggs, causing the myiasis. When the animal finally died, diptera colonization progressed in the carcass until its preservation by refrigeration, and its packaging for necropsy.

## 4. Discussion

Based on the postmortem examination, all the animals included in this study died because of blunt-force trauma. The postmortem findings, combined with the animal’s history of being found near or under overhead lines, support collision with an overhead line.

Different patterns of trauma have been described and related to various sources of trauma. Lesions due to collisions with aircraft, automobiles, and windows are reported in the literature [25,26]. Still, there are scarce reports detailing the traumatic pattern of collisions with overhead lines. Early reports described wing fractures and head trauma in Passeriformes, as well as extremity amputations in small Charadriiforms who collided with lines [27]. Although we only observed postmortem extremity amputation in one predated animal (Case 8), the lack of extremity amputations may be explained by the houbara bustard’s robust skeleton and slower flight compared to Charadriiforms.

In one study of cranes from Japan [5], it was observed that birds who experienced overhead line collisions suffered more commonly from fractures of legs and wings, but sternal, costal, coracoidal and vertebral fractures were also reported. The 29% of the animals suspected to die due to overhead collision experienced more than one bone fracture. The large wings and long, thin legs of cranes were noted to be the most susceptible body parts for this type of traumatism. Miller et al. (2010) [28] reported different “leg problems” in whooping cranes (*Grus americana*), which included leg fractures and hip luxation related to collision. The houbara bustards have shorter and more muscular pelvic limbs, which may explain why we only reported one leg fracture and a knee luxation. Moreover, no head trauma was reported in our study, nor by Takase et al. (2020).

Collision with overhead lines was determined to be a relevant cause of mortality in California condors (*Gymnogyps californianus*) [29]. The authors diagnosed collision with power lines in cases where animals presented compatible traumatic injuries and a history of being found in the immediate vicinity of power lines. In another study on the causes of mortality in raptors, the authors reported a 1.1% incidence of trauma with power lines as the cause of death, but did not report detailed information about the traumatic lesions [30]. Fanke et al. (2011) [31] reported an incidence of 23.4% of mortality due to power line collision regarding Eurasian cranes (*Grus grus*), with trauma along the shoulder girdle, chest, or long bones.

Seven animals in this study carried a GPS transmitter attached to the back with elastic bands. Chronic lesions associated with the transmitter or the harnesses could not be detected in any of them. However, acute lesions, including hemorrhages, bruising, and lacerations, were noted to coincide with the elastic bands of the transmitter in two cases, which raises the question: could these animals collide because of the GPS? It should be mentioned that other studies reported a relationship between birds carrying transmitters and those who collide [28,32,33], but Alonso et al. (2024) monitored a total of 51 Canarian houbara bustards with a GPS, of which only 7 collided. To us, it is uncertain if the transmitter predisposed these animals to collision with overhead lines, or if the corpses were more easily found, submitted to necropsy, and linked with the line because of the transmitter information.

Fibrinogen is produced by hepatocytes and released in the plasma during stress response, and can be detected in degenerated myocytes. In dogs and mice, the immunohistochemical expression of fibrinogen is greater at more than 60 min after trauma [34]. We reported fibrinogen in the hepatocytes forming intracytoplasmic vacuoles in Case 9. Also, we observed the intra- and extracellular globular accumulation of myoglobin and sarcoplasmic fibrinogen deposition, which confirms severe acute muscle degeneration in Cases 6 and 7. These results prove that the animals survived initial trauma for some time, which we estimate to be from minutes to hours [21,34,35]. There are many studies about the formation of fibrinogen in avian species. Still, most of them are focused on the inflammatory response of different infectious agents [36]. In contrast, information about the timing of fibrinogen formation after traumatic injuries in avian species is limited and merits further studies.

When traumatic mortalities are investigated in avian species, most of the studies focus on gross lesions, and little is mentioned about complementary studies that may provide information about survival after the initial trauma [2,3,4,5,6,27,28]. In one study, the authors determined that 36% of whooping cranes (*Grus americana*) that collided with overhead lines survived the trauma [28], whereas Bech et al. (2012) [37] demonstrated that rock ptarmigan hen (*Lagopus muta*) can fly up to 600 m after collision. In our cases, the histopathology, immunohistochemistry, and entomology support that 44.4% (*n* = 4) of animals survived the trauma but died shortly after, due to internal bleeding following an uncertain period ranging from a few minutes to hours (even days, such as in Case 5). It has been reported that birds who collide may survive and have another collision [28]. In Case 3, a healed keel fracture was reported, suggesting that this animal could have collided previously and survived the trauma.

**Figure 5:**
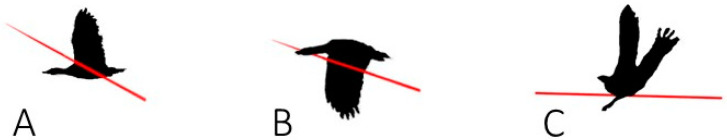
Suggested possibilities for the collisions of the houbara with the lines: (**A**,**B**) The houbara collides with the wings in extension, either dorsally (**A**) or ventrally (**B**). (**C**) The houbara collides with the pectoral or inguinal region during the evasion maneuver.

It has been reported that “poor” fliers and birds with nocturnal flight behavior are more prone to overhead line collisions [8,9]. Moreover, two species of bustards from Spain, the great bustard (*Otis tarda*) and little bustard (*Tetrax tetrax*), had the highest record for collision casualties in a study that included 52 different species [9]. It should be highlighted that the houbara bustard has a crepuscular behavior and is heavier than other birds, which surely predispose them to collide with overhead lines. To explain the lesions observed, we hypothesize that the houbara may collide with the line as depicted in Figure 5. The animal can hit the line while flying with the wing extended dorsally (Figure 5A) or ventrally (Figure 5B). In that case, the trauma may affect the wings, as observed in Cases 2, 7, and 9. More frequently, the houbara may detect the line at the last moment and try to avoid it, resulting in chest, inguinal or leg trauma during the evasion maneuver (Figure 5C), as we detected in Cases 1, 3, 5, 6 and 8.

It should be noted that secondary traumatism may occur, produced by the impact against the ground after the collision with the line, so the coexistence of various patterns of trauma may be expected. The main reported lesions in waterfowls that collide against the ground were liver rupture, hemocoelom and pulmonary hemorrhages, and occasional sternum and ribs fractures [38]. We observed hemocoelom in Cases 4, 6 and 9, but we cannot confirm if the internal hemorrhages resulted from the trauma with the line, from subsequent traumatisms after landing, or even due to the predation of an injured bird.

## 5. Conclusions

We found evidences that the houbaras who collided with overhead lines have characteristic traumatic patterns, and die soon after the collision, within minutes to hours. The histopathology, immunohistochemistry, or entomologic analysis may be helpful to approximate the timing interval between trauma and death.

This study is limited due to the low number of individuals necropsied; however, it contributes to increasing the knowledge in forensic veterinary sciences.

## Figures and Tables

**Table 1:** Location and frequency of the traumatic lesions.

Case Nº	Inguinal Trauma	Skin Laceration	Chest Trauma	Wing Trauma	Hemocoelom	Shoulder Trauma	Neck Trauma	Join Luxation	Head Trauma
1									
2	x	x		x			x		
3	x	x	x						
4	x	x			x	x			
5	x	x						x	
6			x		x	x	x	x	
7	x			x			x		
8	x		x						
9	x	x	x	x	x	x			
%	77.7	55.5	44.4	33.3	33.3	33.3	33.3	22.2	0

## Data Availability

The data presented in this study are available on request from the corresponding author due to legal reasons.

## Supplementary Materials

The following supporting information can be downloaded at: https://www.mdpi.com/article/10.3390/vetsci11070287/s1, Table S1: Summary of the immunohistochemical methodology used in this study.
