# Supplementary material for: Blunt Force Trauma in the Canarian Houbara Bustard (Chlamydotis undulata fuertaventurae) Produced by Collision with Overhead Lines
Source: Vet Sci. 2024 Jun 27;11(7):287. doi: 10.3390/vetsci11070287 (PMC11281538; doi:10.3390/vetsci11070287)
Supplement: Supplementary file 1 [file vetsci-11-00287-s001.zip › vetsci-2996933-supplementary.pdf]

Table S1. Summary of the immunohistochemical methodology used in this study.

ANTIGEN RETRIEVAL	SERUM	SOURCE	DILUTION	PRIMARY ANTIBODY	SOURCE	HOST	TYPE	DILUTION	SECONDARY ANTIBODY	DILUTION	ABC COMPLEX	DETECTION
Citrate buffer (1)	Swine serum (2)	Dako (3)	10% (4)	Myoglobin (5)	Abcam (7)	Rabbit	Polyclonal	1 in 200 (8)	Polyclonal Swine Anti-Rabbit Immunoglobulins (10)	1 in 200 (11)	Vectastain (12)	AEC (13)
Citrate buffer (1)	Swine serum (2)	Dako (3)	10% (4)	Fibrinogen (6)	Abcam (7)	Rabbit	Polyclonal	1 in 50 (9)	Polyclonal Swine Anti-Rabbit Immunoglobulins (10)	1 in 200 (11)	Vectastain (12)	AEC (13)

Detail of the immunohistochemical protocol used in this study:

(1) Citrate buffer, pH 6.0, 10 minutes at 95ºC.

(2) Dako Swine serum (Normal) (X090110-8).

(3) Dako (Glostrup, Denmark).

(4) Dilution of 10 µl of serum in 90 µl of PBS and incubated in a humidity chamber for half an hour to block hydrophobic background staining.

(5) Anti-Myoglobin antibody (ab187506).

(6) Anti-Fibrinogen antibody (ab34269).

(7) Abcam (Cambridge, United Kingdom).

(8) Dilution of 1 µl of antibody in 199 µl of serum at 1% in PBS and is incubated in a humidity chamber for at least 18 hours, inside the refrigerator.

(9) Dilution of 1 µl of antibody in 49 µl of serum at 1% in PBS and incubated in a humidity chamber for at least 18 hours, inside the refrigerator.

(10) Dako Polyclonal Swine Anti-Rabbit Immunoglobulins/Biotinylated (E035301-2).

(11) Dilution of 1 µl of antibody in 199 µl of serum at 1% in PBS and incubated in a humidity chamber for half an hour.

(12) Vectastain® ABC-Peroxidase kit. Vector Laboratories, Newark, US.

(13) 3-amino-9-ethylcarbazole (AEC) and H_2O_2 (0.3%) for 8 min.
